# Inappropriate Shocks With Subcutaneous Implantable Cardioverter-Defibrillator in a Young Patient: A Case Report

**DOI:** 10.7759/cureus.34492

**Published:** 2023-02-01

**Authors:** Zahid Khan, Dinesh Sethumadhavan, Tom Rayner, Sithu Kyaw

**Affiliations:** 1 Acute Medicine, Mid and South Essex NHS Foundation Trust, Southend-on-Sea, GBR; 2 Cardiology, Barts Heart Centre, London, GBR; 3 Cardiology and General Medicine, Barking, Havering and Redbridge University Hospitals NHS Trust, London, GBR; 4 Cardiology, Royal Free Hospital, London, GBR

**Keywords:** hypertrophic cardiomyopathy, scd: sudden cardiac death, apical hypertrophy, loop recoder, icd shock, infective endocarditis, icd storm, ventricular dysrhythmia, implantable cardioverter-defibrillator, subcutaneous implantable cardioverter

## Abstract

Implantable cardioverter-defibrillators (ICDs) are increasingly used for the primary and secondary prevention of sudden cardiac death (SCD). Currently, transvenous (TV) and subcutaneous (S) ICDs are the two different types used. Preservation of central venous vasculature, no risk of vascular or myocardial injury during implant, easier explantation, and lower risk of systemic infections have driven the increased use of S-ICDs. The shocks delivered by ICDs for non-life-threatening arrhythmias or because of oversensing T waves or noise are known as inappropriate shocks. Here, we present the case of a 33-year-old man who had an S-ICD implanted in 2019 for hypertrophic cardiomyopathy. He had a TV-ICD implanted in 2010 which was explanted in 2013 due to infective endocarditis, and the patient underwent a mechanical mitral valve replacement. He was at intermediate risk for SCD over the next five years. He had an S-ICD implanted in 2019 and had never received any shock before. Electrocardiogram showed normal sinus rhythm, left axis deviation, QRS 110 ms, hyperacute T waves in inferior leads, and T-wave inversion in lateral leads. He then began experiencing inappropriate shocks three years after S-ICD placement due to a drop in R wave amplitude secondary to noise oversensing in October 2022. Despite reprogramming the device from the primary vector to an alternate vector, the patient had further inappropriate shocks two months later due to noise oversensing. The patient was discussed in a multidisciplinary team meeting and the S-ICD was explanted according to the patient’s wishes and a loop recorder was implanted.

## Introduction

Ventricular fibrillation (VF) is a common cause of sudden cardiac death (SCD), and various studies have demonstrated the effectiveness of implantable cardioverter-defibrillators (ICDs) in the prevention of SCD [1,2]. SCD commonly occurs due to ventricular arrhythmias in patients with impaired left ventricular systolic dysfunction or myocardial scarring secondary to previous myocardial infarction, myocarditis, amyloidosis, sarcoidosis, and arrhythmogenic ventricular cardiomyopathy (AVC), and ICDs are commonly implanted to treat any life-threatening arrhythmias [3]. The description of ICD shocks can vary, and patients often describe these as the feeling of an earthquake, being hit by a truck, or being kicked by a mole [3]. Transvenous (TV) and subcutaneous (S) ICDs are the major types of defibrillators used for both primary and secondary prevention. S-ICDs are being more commonly used in comparison to transvenous ICDs due to their, advantages such as preservation of central venous vasculature, no risk of vascular or myocardial injury during implantation, easier explantation, and lower risk of systemic infections [3]. The Antiarrhythmics versus Implantable Defibrillators (AVID) trial based on the implantation of ICD for secondary prevention reported that the proportion of patients with an arrhythmic event, which could be defined as SCD, sustained ventricular arrhythmia, or ICD therapy, was 35% at three months, 53% at one year, and 68% at two years [3]. The shocks delivered by ICDs for non-life-threatening arrhythmias or because of oversensing are called inappropriate shocks [4]. This study presents a case of inappropriate S-ICD shocks in a 33-year-old gentleman three years after implantation due to T-wave oversensing.

## Case presentation

**Figure 1:**
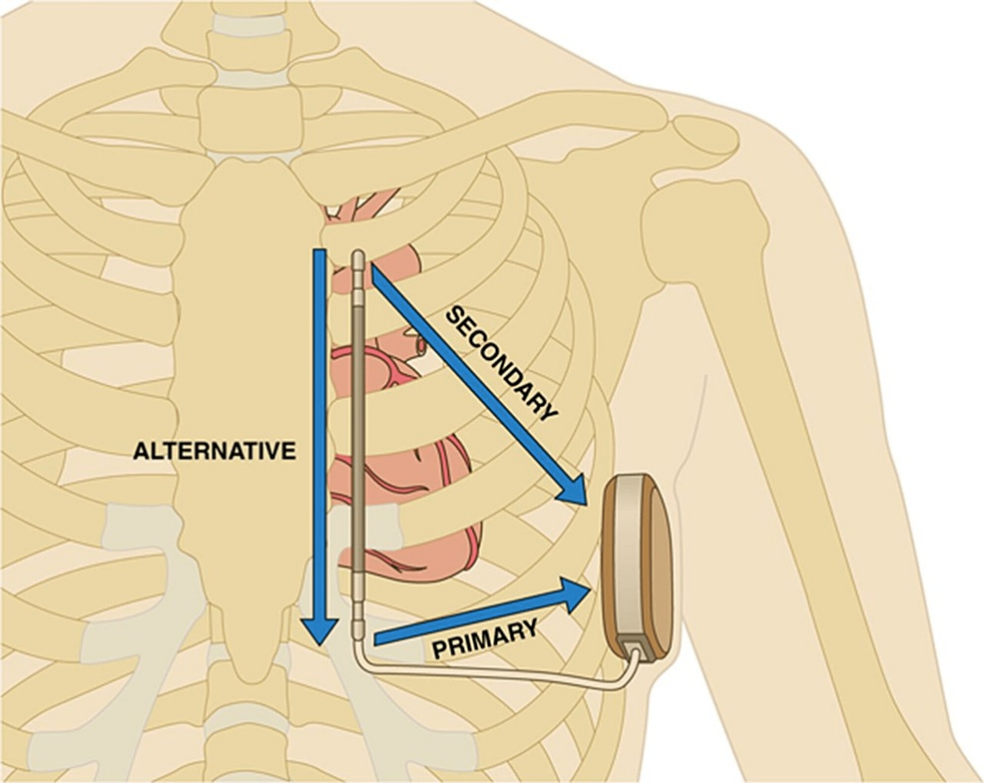
The position of S-ICD vectors on initial screening. S-ICD = subcutaneous implantable cardioverter defibrillator [5].

We present the case of a 33 year-old-man, with a medical history of familial hypertrophic cardiomyopathy (HCM) (MHYZ pathogenic variant), a TV-ICD implanted in 2010 for primary prevention, mechanical mitral valve replacement (MVR) (2013), and explantation of the TV-ICD due to infective endocarditis with suspected lead vegetation. He remained under cardiology follow-up and had a Boston Scientific S-ICD implanted in 2019 due to a five-year intermediate risk of 3.8% for SCD secondary to arrhythmia. Regular medications were warfarin for metallic MVR, bisoprolol 10 mg once daily (OD), ramipril 2.5 mg OD, and sertraline 50 mg OD. He was transferred to our hospital for inappropriate shocks from the device. A device check revealed a drop in R-wave amplitude leading to oversensing and inappropriate shock. All his laboratory tests were normal, and chest radiography demonstrated a slightly lateral placed device without any obvious change in its position compared to the initial implantation. The device check also demonstrated significant noise detection leading to device oversensing resulting in inappropriate shocks, and the noise seemed to disappear immediately after the shock. Electrocardiogram (ECG) showed sinus rhythm with a heart rate of 49 beats/minute, left axis deviation (LAD), QRS width of 110 ms, hyperacute T waves in inferior leads, and T-wave inversion (TWI) in lateral leads. The device was reprogrammed to an alternate vector at 2× gain following a discussion with the representative from Boston Scientific. The patient’s initial S-ICD screening from 2019 demonstrated primary vector failure but passed left-sided alternate vector and right-sided secondary and alternate vectors. He underwent a repeat S-ICD screening in October 2022 after the initial inappropriate shocks that showed significant change from his screening report in 2019 as he passed alternate vector this time only and failed both primary and secondary vector screening whereas he passed both secondary and alternate vectors in 2019 but failed primary vector (Tables 1, 2). The position for these vectors is shown in Figure 1. The requirement for minimum screening criteria was satisfactory readings for at least one lead in all tested postures.

**Table 1:** Initial screening prior to S-ICD implantation in 2019 shows satisfactory function in secondary and alternate leads. S-ICD = subcutaneous implantable cardioverter defibrillator

Sternal lead position	Sternal lead summary							
Lead	Supine	Standing/Sitting	Original implant location	Implant post standing	Stretch	Optional posture	Morphology consistent between postures?	Mark all acceptable leads
Primary lead – III	Fail	Okay	Fail	Okay	Fail	-	Yes ✓ No	
Secondary lead – II	Fail	Fail	Fail	Fail	Fail	-	Yes ✓ No	✓
Alternate lead – I	Okay	Okay	Okay	Okay	Okay	-	Yes ✓ No	✓

**Table 2:** S-ICD screen after the initial shock shows satisfactory function in the alternate lead only. S-ICD = subcutaneous implantable cardioverter defibrillator

Sternal lead position	Sternal lead summary							
Lead	Supine	Standing/Sitting	Original implant location	Implant post standing	Stretch	Optional posture	Morphology consistent between postures?	Mark all acceptable leads
Primary lead – III	Fail	Okay	Fail	Okay	Fail	-	Yes ✓ No	
Secondary lead – II	Fail	Fail	Fail	Fail	Fail	-	Yes ✓ No	
Alternate lead – I	Okay	Okay	Okay	Okay	Okay	-	Yes ✓ No	✓

**Figure 2:**
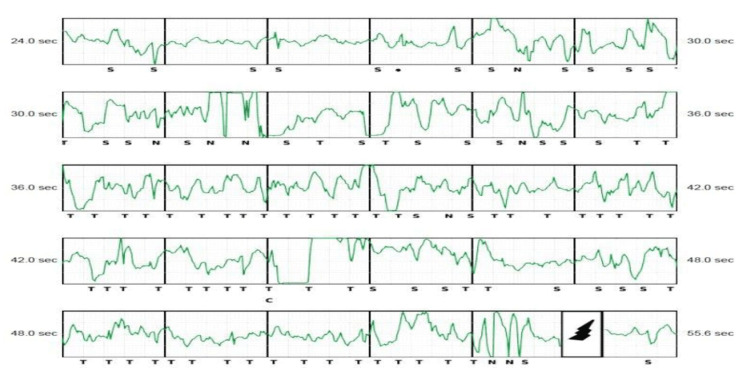
EGM tracing showing noise and subsequent shock therapy. EGM = electrogram

**Figure 3:**
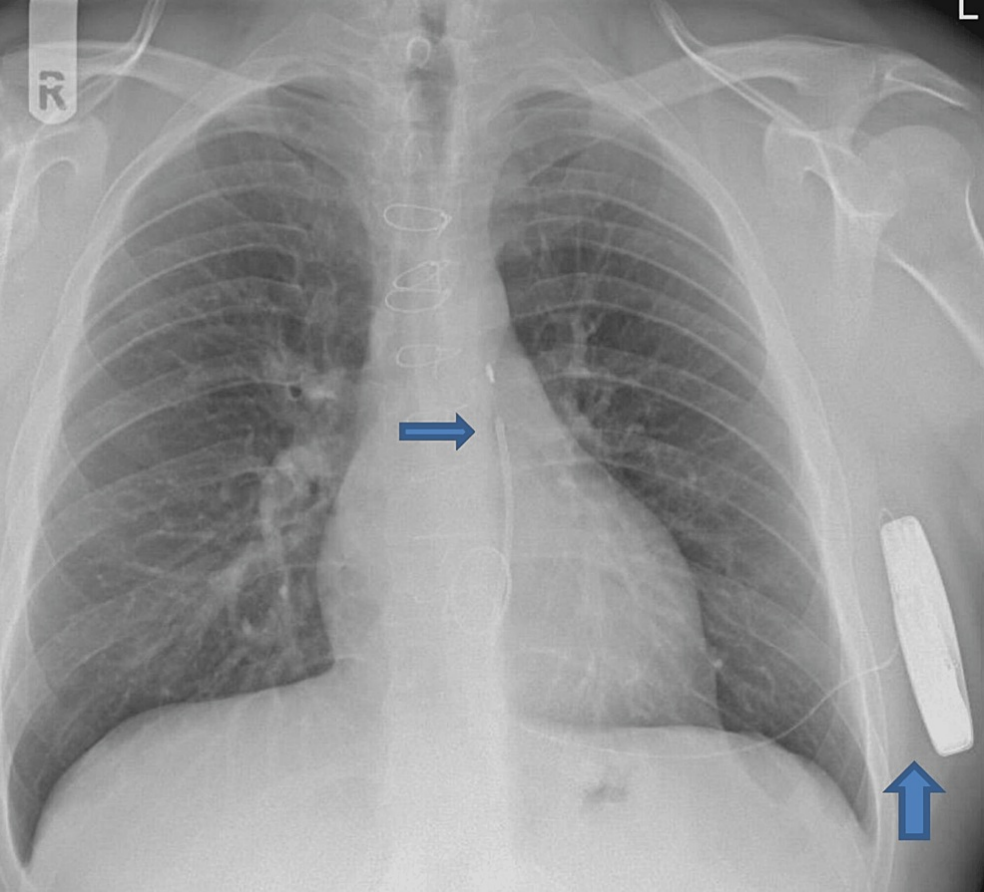
Chest radiography AP view showing the ICD in situ (pointed arrow). ICD = implantable cardioverter defibrillator; AP = anteroposterior view

**Figure 4:**
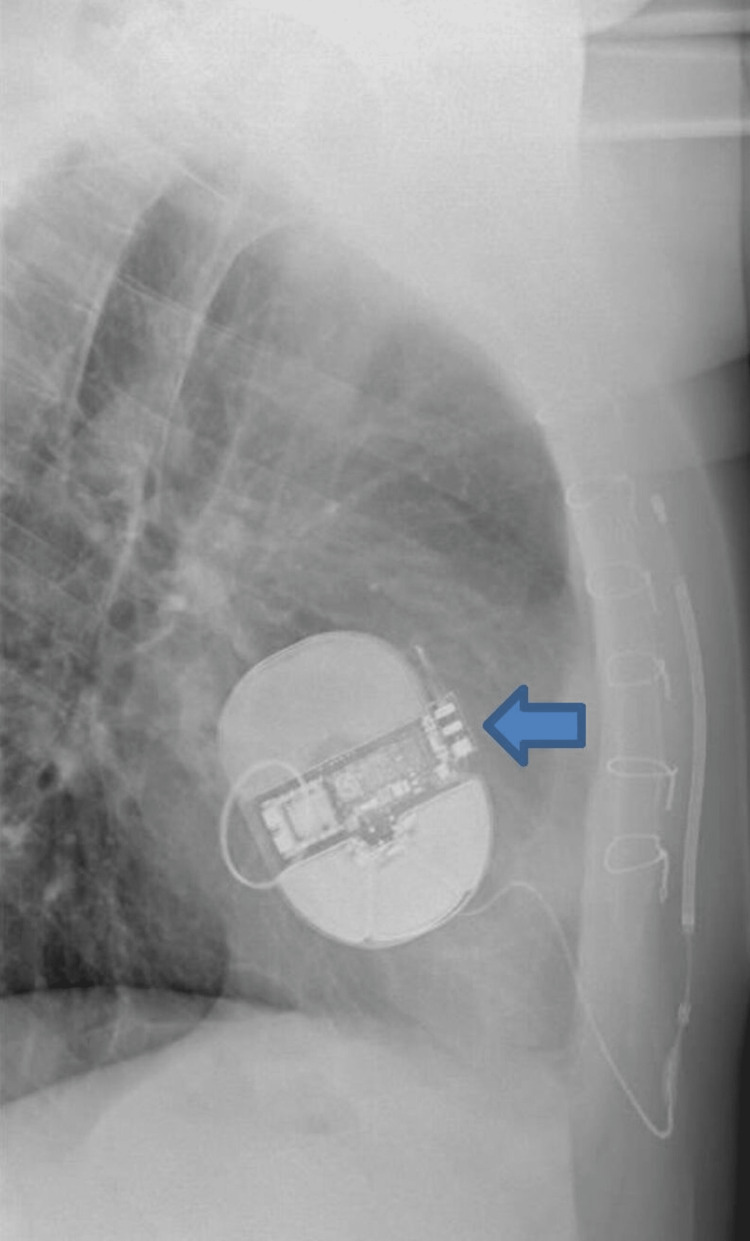
Chest radiography lateral view showing the ICD (pointed arrow). ICD = implantable cardioverter defibrillator

The patient was discussed in a multidisciplinary team (MDT) meeting, and in view of his SCD risk being intermediate, it was suggested to discuss with the patient the options of the device off vs. extraction vs. device on vs. reimplantation of the TV device. After a discussion with the patient who himself felt unprotected without the device, device therapy was left on and the device was reprogrammed with a range changed to 2 with the best R-to-T-wave ratio seen on all vectors, and the vector was changed from primary to alternate. The patient also felt traumatized from the shock and was reviewed by a psychologist. After two months, he presented to the accident and emergency department in his local hospital with further shocks from the S-ICD when he was sleeping. A device check revealed inappropriate shocks because of noise oversensing, and, subsequently, the therapies were turned off (Figure 2). The patient was transferred to our hospital for further management. Chest radiography showed no change in the lead positions since implantation (Figures 3, 4).

His observations were stable, ECG remained unchanged with sinus rhythm and a heart rate of 55 beats/minute, left axis deviation, QRS of 119 ms, hyperacute T waves in the inferior leads, and TWI in the lateral leads. His blood results were normal with a potassium of 4.3 mmol/L and magnesium of 0.9 mmol/L. His echocardiogram showed mildly impaired left ventricular ejection fraction (LVEF) (45- 50%), normal left ventricle with asymmetrical hypertrophy, and mildly dilated left atrium, and mechanical MVR appeared well seated with a trivial leak, similar to previous imaging (Videos 1, 2).

**Video 1:** TTE SAX view showing mildly impaired LVEF. TTE = transthoracic echocardiogram; SAX = short-axis view; LVEF = left ventricular ejection fraction

**Video 2:** TTE LAX view demonstrating mildly impaired LVEF and mMVR in situ. TTE = transthoracic echocardiogram; LAX = long-axis view; LVEF = left ventricular ejection fraction; mMVR = mechanical mitral valve replacement

Cardiac magnetic resonance imaging (CMR) showed normal left ventricle size, mildly hypokinetic septum, and a normal LVEF of 71%. Late gadolinium enhancement was observed in both right ventricle insertion points extending into the basal anterior and inferior septum and asymmetric septal hypertrophy (MWT 29 mm basal antero-septum). It was decided to try optimizing the device programming at the first instance. A device interrogation was performed with the representative from Boston Scientific, and it was found that there was intermittent T-wave oversensing on primary and secondary vectors and significant noise contributing to these inappropriate shocks, following which the case was referred to the MDT again. The patient made his wishes explicitly known that he wanted the device to be explanted. He never received an appropriate shock from either the TV or S-ICD since implantation and his HCM-SCD five-years risk was 3.8% (Table 3). He had S-ICD explanted following discussion in the MDT, and an implantable loop recorder (ILR) was placed to monitor for arrhythmias. He had further follow-up in the device clinic after three months, and ILR checks did not show any evidence of arrhythmia. The patient continues to be followed up in the outpatient clinic.

**Table 3:** HCM-SCD risk for the patient. HCM = hypertrophic cardiomyopathy; SCD = sudden cardiac death; MWT = mitral valve replacement; LVOT = left ventricular outflow tract [6]

Title	Score
HCM-SCD five-year risk	3.8%
Age	33 years
MWT	29 mm
Left atrium	53 mm
LVOT	5 mmHg
Family history of SCD	None
Non-sustained ventricular tachycardia	None
Previous syncope	None

## Discussion

ICDs have been increasingly used in the last two to three decades to minimize the risk of SCD in patients with various cardiac conditions. Transvenous lead placement with a subcutaneous, pectoral pulse generator has been the traditional approach for ICD implantation for the past two decades and has demonstrated remarkable success [5]. Despite the improvement in technology, increased operator experience, and better surgical technique, there are risks associated with TV-ICDs, such as lead fracture, infections, and pneumothorax [5]. The risk of infections is reported to be about 1.5% including at both cardiac and non-cardiac sites. Lead complication rates have been reported to be about 10% in randomized controlled trials, with increasing annual failure rate which is proportional to time after implantation [5,6]. The main reasons for failure include insulation defects, lead fracture, dislodgement, insulation defects, loss of capture, inability to sense appropriately, and abnormal impendence [6,7]. To address these concerns, S-ICD was developed and gained approval in Europe in 2008 and Food and Drug Administration (FDA) approval in 2012 [8,9]. With the increased use of S-ICD implantations over traditional ICDs, there are no data to suggest the superiority of the former over the later with regard to clinical outcomes, although S-ICDs are preferred in patients with venous access issues or infection risk and younger patients [8,9]. One of the major limitations of S-ICDs is its lower programmable parameters, including detection of heart rate for conditional shock, committed shock zones, therapy output, sensing vector, and transient post-shock pacing when compared to TV-ICDs [3].

Although both ICDs have a risk of inappropriate shocks, the incidence associated with S-ICD is reported to be 12-29% [8]. The reported causes for inappropriate discharges include T-wave oversensing, noise from the internal or external source, supraventricular tachycardias (SVTs), oversensing of low-amplitude signals, non-cardiac sensing, subcutaneous air, generator change, and myopotential oversensing [10-13]. Among the above causes, cardiac signal oversensing (73%), including T-wave oversensing and noise, remains the most common etiology for inappropriate shocks in S-ICD [13,14]. The EFFORTLESS S-ICD Registry, which collected S-ICD implantation information and follow-up data from clinical centers in Europe and New Zealand, reported that 48 out of 581 S-ICD patients (71% male, age 49 ± 18 years) experienced 101 inappropriate shocks (8.3%) during the follow-up period of 21 ± 13 months [13]. The most common cause for inappropriate shocks was cardiac signal oversensing such as T-wave oversensing observed in 73% of patients, followed by SVT with 18 (18%) shocks, of which 15 occurred in the shock-only zone. Patients with atrial fibrillation and HCM were noted to be at higher risk for inappropriate shocks, and this risk was reduced by reprogramming the primary vector for sensing (from xiphoid to V6). Similarly, reprogramming the device or optimization of SVT treatment after the first clinical event of inappropriate shock successfully prevented further inappropriate shocks for cardiac oversensing and SVT events [13,14].

In the same study, few patients had device extractions due to anti-tachycardia (n = 5), biventricular (n = 4), or bradycardia pacing (n = 1). The incidence of inappropriate shocks in patients was 8.1% at one year and 11.7% at three years. The frequency of one and five-year appropriate shock therapy was reported as 5.8% and 13.5%, respectively, with overall successful conversion for spontaneous episodes at 97.4% [13]. Our patient was diagnosed with HCM in 2009 and underwent implantation of TV-ICD for primary prevention in 2010 which was later explanted due to infective endocarditis in 2013. Subsequently, he underwent implantation of S-ICD in 2019 after passing the vector screening as he was deemed to have an intermediate risk of arrhythmias. He started receiving inappropriate shocks after three years of implantation due to a drop in R-wave amplitude which was reprogrammed. Despite this, he continued to have inappropriate shocks which were affecting his psychological well-being as well. Further investigations revealed T-wave oversensing and noise resulting in inappropriate shocks, and the S-ICD was explanted.

## Conclusions

With the expanding use of S-ICD for the prevention of SCD, it becomes essential to be aware of the various etiologies of cardiac signal oversensing causing inappropriate shocks, as it can have a negative impact on patient outcomes. Patients should have S-ICD screening prior to implantation of these devices and should receive these devices provided they pass most vectors. It is also important to be aware of causes that can contribute to change in the outcomes of these vectors. These patients should be discussed in multidisciplinary meetings to provide the best possible treatment, and patients with inappropriate shocks also need psychological support in addition to clinical management.
